# Perspective of pediatricians and adolescent patients on the transition process in a university hospital

**DOI:** 10.1590/1984-0462/2022/40/2020490

**Published:** 2022-01-05

**Authors:** Luiza Mariana Cordeiro Silva, Letícia Mansano Souza, Elizete Prescinotti Andrade, Lilia D’Souza-Li

**Affiliations:** a Universidade Estadual de Campinas, Campinas, SP, Brazil.

**Keywords:** Adolescent health, Adolescent medicine, Health care transition, Adolescent health services, Chronic disease, Saúde do adolescente, Medicina do adolescente, Transferência de pacientes, Serviços de saúde do adolescente, Doenças crônicas

## Abstract

**Objective:**

To map the transition process from the perspective of pediatricians and their adolescent patients, and to suggest a transition protocol.

**Methods:**

This is a descriptive, cross-sectional study conducted in a pediatric outpatient clinic of a public tertiary hospital. Pediatricians answered a questionnaire about the transition process, and that was evaluated in a descriptive manner. The Transition Readiness Assessment Questionnaire (TRAQ) on health autonomy was answered by the adolescents and the analysis was performed using the χ^2^ and Mann-Whitney tests. p<0.05 were considered significant.

**Results:**

31 pediatricians (16 residents, 15 supervisors) were enrolled, with a mean age of 40.1 (±16.9), 87% women, with years working in Pediatrics ranging from 2 to 45 years (median of 5 years). Most doctors agreed that there was no transition plan, but they stimulated the patient’s autonomy and talked to the patient and family members about any existing chronic diseases. A total of 102 adolescent patients participated, with a median age of 15; 56% were female. The TRAQ median was 58, with similar scores between females and males, and higher scores in those older than 16 years of age (Mann-Whitney U test, p=0.01). The patients reported ease in face-to-face communication with their doctors, but great difficulty in talking about health issues over the phone.

**Conclusions:**

Even without a transition protocol, adolescents developed several self-care skills as they aged. The lack of a transitional protocol led to conflicting opinions, which reinforces the need for improvement. We suggest a flowchart and transition protocol.

## Resumo

**Objetivo:**

Mapear o processo de transição na perspectiva de pediatras e de seus pacientes adolescentes bem como sugerir um protocolo de transição.

**Métodos:**

Estudo descritivo, transversal, realizado em um Ambulatório de Pediatria de um hospital público terciário. Pediatras responderam a um questionário sobre o processo de transição, que foi avaliado de forma qualitativa. O Questionário de Avaliação do Preparo para a Transição (TRACS) foi respondido pelos adolescentes, e a análise foi feita com testes do qui-quadrado e de Mann-Whitney. Valores p<0,05 foram considerados significantes.

**Resultados:**

Participaram do estudo 31 pediatras (16 residentes, 15 supervisores), com média de idade de 40,1 (±16,9) anos, 87% do sexo feminino, tempo de atuação na Pediatria variando de dois a 45 anos, com mediana de cinco anos. A maioria dos médicos concordava que não havia um plano de transição, mas eles estimulavam a autonomia do paciente e conversavam com pacientes e familiares sobre qualquer doença crônica presente. Participaram da pesquisa 102 pacientes adolescentes, com mediana de idade de 15 anos, 56% do sexo feminino. A mediana do TRACS foi de 58, com escores semelhantes entre os sexos feminino e masculino, e escores superiores nos maiores de 16 anos (teste U de Mann-Whitney, p=0,01). Os pacientes relataram facilidade na comunicação presencial com seus médicos, mas grande dificuldade para falar sobre questões de saúde por telefone.

**Conclusões:**

Mesmo sem protocolo de transição, os adolescentes desenvolveram várias habilidades de autocuidado com o avanço da idade. A falta de protocolo levou a opiniões conflitantes, reforçando a necessidade de melhor estruturação. Os autores sugerem a criação de um fluxograma e um protocolo de transição.

## INTRODUCTION

Transition is the gradual process of preparing adolescents and their families for referral from a pediatric service to adult care, adopting new roles and promoting their independence and autonomy.^1^ The goal of the transition process is to maximize the functioning and potential of the individual throughout life via high-quality and development-appropriate health services that continue without interruptions as they move from adolescence to adulthood.^2,3^ It is a dynamic and complex process that requires planning, stimulation of acquisition of skills and competences, and assessment of the state of readiness of each patient. It differs from the transfer that implies only the act of referring the patient from one institution or care team to another.^2^

Adolescence is a phase of change, when the individual takes on new responsibilities and challenges, laden with conflicting emotions, mourning, contradictions and deconstructions.^4^ When adolescence is experienced while having a chronic disease, the challenges are even greater, as these patients require special health care. Every adolescent wants to belong to a group and to seek uniformity within it, and being different from the group can generate even more stress, resulting in greater vulnerability and risk to the discoveries and trials common in adolescence. Adolescents with chronic diseases are more likely to start risky behaviors, such as unprotected sexual activity and use of substances like alcohol and tobacco,^5^ increasing the complexity of the transition process.^2^

Qualitative research with young adults demonstrated that, in the absence of a transition protocol, these patients experienced the change in the presentation of care in a disjointed manner, showing difficulties in adapting to increased responsibilities and self-care.^6^

Understanding the social context in which the adolescent is inserted is indispensable in order to make a transition from health care, offering support that meets the needs of the patient at that time.^7^ The social impairment that the disease can cause since childhood should be noted, such as days of absence from school, limitations of daily activities and delay in development milestones.^8^

Approximately 20% of Brazilian adolescents present at least one chronic condition,^9^ and, despite huge demand, we found few studies on transition. In the national scenario, there is no standardization of how these processes are performed or evaluated.

This study mapped out the transition process of adolescent patients from pediatric outpatient clinics to adult outpatient clinics, and several variables involved in the process from the perspective of pediatricians and their patients. Based on our findings, we suggest a flowchart and a transition protocol. Seeking for ways to systematize and improve the transition of care for adolescents serves to reduce risks and stimulate their autonomy.

## METHOD

This is a descriptive, cross-sectional study developed in an outpatient Pediatric service in the public university hospital of a metropolitan region. The study was approved by the local Ethics Committee of the State University of Campinas, Certificado de Apresentação de Apreciação Ética (Ethics Appreciation Presentation Certificate) (CAAE): 18727919.4.00005404.

The entire Pediatric medical team working in specialty outpatient clinics was invited to participate. Participants with partially completed questionnaires were excluded. Adolescents aged 12 to 18 years were invited while waiting for consultation in Pediatric outpatient clinics, without distinction of specialties. Patients who had some cognitive impairment and could not answer the questions were excluded.

Data collection took place from September 2019 to January 2020, performed by the first and second authors, preceded by the signing of the terms of free and informed consent and consent to minors.

Sociodemographic data was collected from physicians (gender, age, specialty, how long they have worked in the field, and what role they performed in the outpatient clinic), with subsequent application of a questionnaire developed in the city of Porto in 2012, containing 15 questions (with 3 response options: Yes, No or To be implemented), regarding how the transition process occurred and the stimulation of the adolescent’s autonomy in the outpatient clinics.^1^ This was the only tool found in Portuguese for the purpose of evaluating the transition process from the point of view of the assistance team. The medical questionnaires were analyzed in a descriptive manner.

We applied two questionnaires: to assess how prepared the adolescent patients were regarding self-care and autonomy, TRAQ was used, as well as a sociodemographic questionnaire regarding age, sex, how long the patient was attended to in the outpatient setting (and in which clinic), whether they came to the consultation with company, whether they had a doctor they considered to be a reference, and what their specialty was.

The TRAQ is a self-administered questionnaire that has been translated and validated into Brazilian Portuguese, and includes 20 questions that assess the patient’s skills for self-care and their ability to transition to medical care in the adult clinic.^2^ The questionnaire presents 5 domains: A) Managing medication, B) Appointment keeping, C) Tracking health issues, d) Talking with providers, E) Managing daily activities. The questionnaire is of *likert* type, offering, for each question, 5 options to respond, which we score incrementally from 0 (No, I don’t know how), 1 (No, but I want to learn), 2 (No, but I’m learning to do this), 3 (Yes, I have started doing this) up to 4 points (Yes, I always do this when I need to), with a minimum score of 0 and maximum score of 80. We considered that the higher the score, the fitter the patient was for the transition.

A database spreadsheet was created for analysis. The negative and intermediate responses of the TRAQ questionnaire, that is, when the patient either did not know how to do or did not yet perform the activity, but would like to learn, were condensed into one alternative, and the positive responses into another alternative. For the analysis of categorical data of the TRAQ questionnaire, the χ² test was used, comparing children under 16 years with those older than or equal to 16 years. Because of the distribution by age, TRAQ and follow-up time did not present normal distribution, therefore, the Mann-Whitney test was used to verify association with both age and sex groups. A p<0.05 was considered significant.

## RESULTS

Of the 60 pediatricians invited, two refused to participate and 31 (16 residents, 7 hired doctors and 8 teachers) returned the questionnaire fully filled. Physicians of the following Pediatric specialties participated: Allergy and Immunology, Nephrology, Pulmonology, Gastroenterology and Hepatology, Obesity, Endocrinology and Cardiology. The mean age was 40.1 (±16.9), with 87.1% being female. The length of time in Pediatrics among the interviewees ranged from 2 to 45 years, with a median of 5 years.

Most physicians agreed that there was no transition plan (Table 1, question 2) and that there were no teaching materials available to offer patients regarding their health issues (question 7). Most of them encouraged the patient’s autonomy (question 5), were able to exchange information with other doctors who accompanied the patient (question 10), and talked with the adolescents and family about their illnesses (question 11a). The privacy aspect of the adolescent’s care was discordant between the residents and the supervising doctors (teachers and contractors): the residents had the opinion that care was done with escorts, while most of the supervisors replied that it was without escorts.

**Table 1.** Questionnaire applied to physicians showing the percentage of responses for each item that occurred or did not occur in outpatient clinics. The answers in which at least 80% of the participants agreed are in bold.

Questions	Yes	No	To be implemented
1. Is there a professional or a team of health professionals dedicated to the transition process?	14 (45%)	16 (51%)	1 (3%)
2. Is the transition plan implemented during early adolescence (11–14 years) and subsequently revised with the adolescent between 14–18 years?	**1 (3%)**	**27 (87%)**	3 (9%)
3. Are adolescents encouraged to intervene in the transition process?	9 (29%)	22 (71%)	0
4. Before the transfer, are there joint consultations between the pediatrician and the adult health care doctor?	10 (32%)	20 (64%)	1 (3%)
5. Is the adolescent stimulated to have knowledge about their pathology and to develop skills that incite their autonomy?	**25 (80%)**	**5 (16%)**	1 (3%)
6. Does the adolescent have the opportunity to go to consultations without their parents?	16 (51%)	14 (45%)	1 (3%)
7. Are materials such as books or magazines that talk about the difficulties encountered by young people with chronic pathologies provided?	**2 (6%)**	**29 (93%)**	0
8. Is there a concern about ensuring that, during the transition, multidisciplinarity is maintained so that there is cooperative work between all the components of the team (pediatrician, adult health care doctor, social worker…)?	12 (38%)	16 (51%)	3 (9%)
9. Is there a concern about involving the patient’s referral physician in this process?	12 (38%)	18 (58%)	1 (3%)
10. Is there ease of access to medical information among the doctors who treat the adolescent?	**25 (80%)**	**6 (19%)**	0
11. During the transition, are:			
11 a. Health problems discussed so that the family and the adolescent understand the disease?	**25 (80%)**	**5 (16%)**	1 (3%)
11 b. Changes in roles between young people and family discussed?	20 (64%)	9 (29%)	2 (6%)
11 c. Social, psychological (including self-esteem), communication and sexual issues discussed?	20 (64%)	9 (29%)	2 (6%)
11 d. Plans for the future (academic and vocational training) defined?	23 (74%)	7 (22%)	1 (3%)
11 e. Differences between adult healthcare and pediatric service mentioned?	20 (64%)	10 (32%)	1 (3%)
11 f. Visits to the adult healthcare service and their scheduling provided?	10 (32%)	21 (67%)	0

Some of the professionals reported that only a few outpatient clinics with specific groups of patients presented a structuring of the transition process, such as in the Immunology HIV outpatient clinic. In their opinion, the transition could be hampered or even prevented by the absence of a receiving physician in the adult clinic, with skills in managing the particularities of some diseases (e.g. genetic syndromes, such as cystic fibrosis or sickle cell anemia). The bond created with the pediatric service was so strong that some adult patients — with follow-up since childhood or adolescence — could not disconnect from the service and refused to be transferred to the care of the medical clinic. The medical team realized that some parents had difficulty stimulating the autonomy of their adolescent children and resisted giving up responsibility for all aspects of care.

The TRAQ questionnaire was applied in 102 patients in the Pediatric waiting room. None of the invited patients refused to participate, and no questionnaires were excluded. The median age was 15 years, 56% were female; regardless of the length of time of follow-up in the outpatient clinic, only 50% had a referral doctor, whom they turned to when they had any health issue (Table 2). A small fraction of the patients (6%) went without escorts to consultations, and all were over 16 years and female. The median TRAQ score was 58, with similar scores between female and male (Mann-Whitney U test, p=0.348). Gender did not affect self-care, except for the management of daily activities, in which the female adolescents had higher scores. To check if older adolescents presented more autonomy, we separated the participants into two groups according to age: those under 16 and those with 16 years or more. The median of TRAQ scores was higher in older adolescents (Mann-Whitney U test, p=0.01). The main differences between the groups were in the domains of medication management and consultations (Table 2), with greater proportion of older adolescents performing these activities.

**Table 2.** General profile of adolescents monitored in pediatric outpatient clinics, separated by age — either younger or older than 16 years — and frequency of positive responses in the Transition Readiness Assessment Questionnaire (“Yes, I have already started doing this” or “Yes, I always do this when I need to”). A questions are part of medication management, B of consultation management, C of monitoring of health problems, D of communication with health professionals, and E of management of daily activities.

Characteristics and questions	<16 years	≥16 years
Patients (n=102)	54	48
Female (n=57)	31 (56%)	26 (55%)
Has referring doctor (n=51)	29 (52%)	22 (46%)
With escort during consultations (n=96)	54 (100%)	42 (89%)*
Median follow-up time (4.5 years)	3	6*
Median Transition Readiness Assessment Questionnaire score (58)	54	62*
A1 Brings a prescription to the pharmacy to purchase medications	47 (85%)	42 (89%)
A2 Knows what to do if you have side effects to medications	29 (52%)	34 (72%)*
A3 Takes medication by themselves and correctly	38 (69%)	39 (83%)
A4 Gets more medication before it runs out	40 (74%)	42 (89%)*
B1 Makes calls to schedule consultations	30 (54%)	38 (82%)*
B2 Follows referrals for laboratory tests	44 (81%)	45 (95%)*
B3 Arranges their own transportation for going to consultations	36 (65%)	42 (89%)*
B4 Calls the doctor to talk about health issues	4 (7%)	13 (27%)*
B5 Gets a health plan if the current coverage is lost	18 (33%)	17 (36%)
B6 Is able to tell whether their coverage is public or private	40 (72%)	35 (74%)
B7 Manages money and/or budget household expenses	9 (16%)	15 (32%)
C1 Fills out medical history and allergy list forms	28 (51%)	33 (70%)*
C2 Has a schedule or list of medical consultations and other appointments	29 (52%)	38 (84%)*
C3 Devises a list of questions or doubts before going to the doctor	27 (49%)	30 (63%)
C4 Has financial help to study or work	52 (96%)	38 (81%)*
D1 Tells the doctor or nurse what you are feeling	52 (94%)	45 (98%)
D2 Answers questions asked by medical staff members	53 (100%)	45 (98%)
E1 Helps prepare or plan meals	33 (60%)	28 (59%)
E2 Keeps the house or room clean or helps in cleaning	40 (72%)	41 (87%)
E3 Uses neighborhood shops and services	53 (96%)	44 (93%)

* p<0.05, χ^2^ test and Mann-Whitney test.

The questions that the adolescents most marked as “No, I do not know how” were about appointment keeping, items B4 and B7, which corresponded to “Call the doctor to talk about health issues” and “Manage your money or having some home budget”, respectively. During the application of the questionnaires, the adolescents and their parents were interested in learning more about these items. The questions that adolescents, regardless of age, mostly answered “Yes, I always do this when I need to”, were those regarding face-to-face communication with health professionals.

## DISCUSSION

We identified no transition protocol in the outpatient clinics of Pediatric specialties of the institution studied. Despite this, there was communication between the different attending physicians and encouragement of patient autonomy.

The transition constitutes an important milestone for the doctor-patient relationship and for the continuity of follow-up, being pointed out by several studies as a critical moment for loss of bond, abandonment of follow-up, and complications related to the underlying disease.^4^

In addition to the supervising physicians, resident physicians from several Pediatric specialties participated in our study, which broadened the perspective of care. We did not find data that differentiated the level of training of the participants, or training time in Pediatrics in other studies about transition.

In general, the opinion of the resident doctors and supervisors aligned, except in regard to the performance of the adolescent’s consultation in two stages (one part without guardians). This difference may suggest a different view of the residents regarding management of adolescents, in addition to a non-uniformity of care. The non-standardization of the care model is constant in studies conducted in places that do not have a transition protocol.^2,4,5^

The participating physicians perceived in some parents a certain difficulty in stimulating the self-care of children who suffer from chronic diseases, a frequent issue in several studies. Stinson and colleagues observed that overprotective parents represented an obstacle to transition, and their teenage children showed less interest in knowing and managing their underlying disease. In order to encourage autonomy, the authors reinforced the importance of medical care without parents, and the inclusion of patients in decisions that concern their health.^10^

The transition of care is not a much discussed topic in medical consultations: Van Staa et al.^11^ state that 65.3% of adolescents with chronic diseases reported never having discussed the topic, and only 7.4% had frequent discussion; the group that included more discussion on the matter presented a better level of readiness.

Differences between genders in TRAQ were identified only in the domain of management of daily activities, especially in domestic activities. Similar results were observed in other studies, suggesting that girls perceived their abilities in these areas as more developed, perhaps due to social constructs.^12^

In a Brazilian study by the Hematology and Hemotherapy Foundation of Pernambuco (HEMOPE), using its own questionnaire, only 50.8% of adolescents were responsible for the regular use of medications, and 77.8% did not know how to schedule appointments and needed help.^13^ In contrast, in our study, 84% of adolescents wrote down their medical consultations or other appointments, and 83% of adolescents between 16 and 18 years of age were able to call to schedule consultations. Although face-to-face communication was considered good, most reported great difficulties in talking about health issues over the phone. Our research was conducted in a period prior to the COVID-19 pandemic; in the new health scenario, face-to-face care in outpatient clinics was discontinued. Since patients already had difficulty talking about their health problems over the phone, we questioned whether their health needs are being met.

The adolescents and parents were interested in learning more about the items marked “I don’t know how to do this” in TRAQ, which shows opportunity for discussion of the topic and interventions to improve quality of life. The adolescents developed several self-care skills, which may be associated with environmental stimuli, family or individual search for knowledge.

Due to the lack of a transitional protocol, there were conflicting opinions as to what was or was not done, which reinforces the need for better structuring. The fact that professionals perform some transition measures may suggest that there is room for discussion and systematization of this process.

As limitations of this study, the clinician sample was purposively selected, but only half of the clinical staff returned the questionnaire. Thus, our results may not reflect the conduct and opinions of all the professionals working in the outpatient clinic. There is also the consideration that the study was conducted in an outpatient clinic of a tertiary hospital, so the results may not reflect all aspects of patient care. The patients were selected from a convenience sample and their opinion may not reflect the general patient perception.

**Figure 1.**
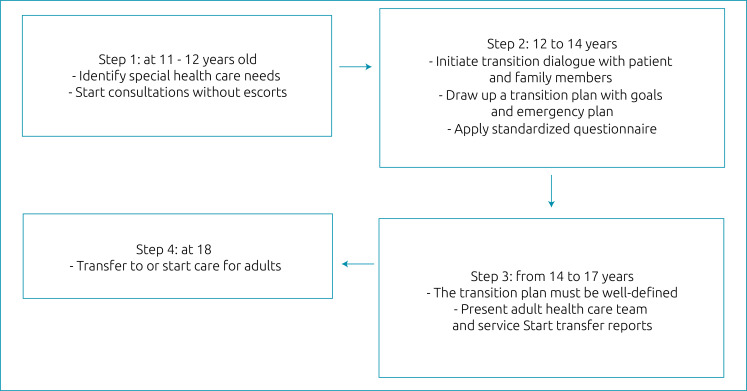
Transition flowchart.

The transition goes far beyond a checklist or a conversation: it is a continuous and adaptable process, with needs that change according to the maturity of the patient. The Brazilian Society of Pediatrics issued a document on transition; it stresses the importance of an organized process and discusses the roles of family members, the health team and adolescents during this period.^14^ In order to systematize and improve the transition of care for adolescents, we suggest following a protocol for transition (Figure 1).

During the whole process of monitoring the patient, whether healthy or with some pathology, it is important to stimulate an open dialogue with them and their family members, as well as to use accessible terms and materials to clarify what they present or may come to present. The steps below are suggestions of an organized flow for transitioning, but it is important that the individuality of the patient is taken into account to devise their plan.

The transition plan is part of the adolescent’s medical record and should contain the start date, goals and how to achieve them, the dates of each step and the result of standardized scales that include: adolescent routine, self-care, medication management, communication with the health team and care to the environment where they live. It should be continuously revised and updated as needed by the patient. Stratification of the process makes it easier to understand what occurs in each period. It is important to assess the level of understanding of the teenager about what will be discussed or stimulated.

**Figure 2.**
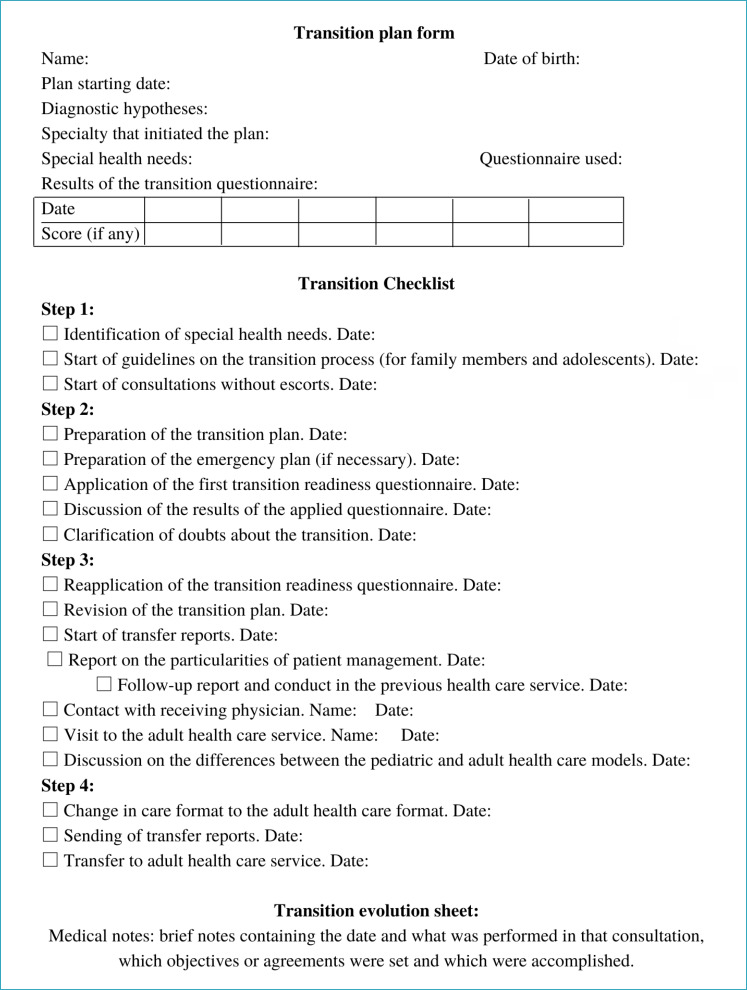
Example of a transition plan form to guide the physician during the process.

Figure 2 suggests a transition plan form, containing a checklist of items to be performed in each step and an evolution sheet. The ages indicated may change according to the adolescent’s health status, and patients with more severe conditions may require an anticipation of the stages of the transition process.

- Step 1 (11–12 years): involves the identification and evaluation of possible particularities or health needs, as well as the beginning of consultations without escorts. At this time, the clarification of what the transition process is begins.
- Step 2 (12–14 years): involves new discussion with teenagers and their family about what the transition is and what its goals are, creation of the transition plan in joint manner (with patient and family), application of a standardized questionnaire and the setting of goals and deadlines for the plan’s items. We also suggest drawing up an emergency plan whenever necessary. This plan should contain, in addition to the diagnosis and the evolutionary stage in which the disease is, what measures should be carried out when the adolescent needs to go to the emergency room, as well as provide possible contacts to ask for clarification.
- In Step 3 (14–17 years), the transition plan should already have been revised and adapted a few times, and should be well-established. Initiate the preparation of transfer reports and communication with the doctor who will continue the patient’s care, in the most feasible way (e-mail, face-to-face or via telephone), providing documents and important information. The transfer report should contain information about the adolescent’s disease, their particularities, frequent causes of decompensation, possible comorbidities, as well as therapeutic measures carried out during the period of follow-up in previous Pediatric services. Between the ages of 16 and 17, we suggest a visit of the adolescent to the adult healthcare service, ideally accompanied by someone from the Pediatric team, showing the future place of care and the team that will serve them. If the adolescent remains with the Pediatric team, the differences present in adult health care must be discussed.
- In Step 4, at the age of 18, the type of care is changed (if the patient continues with the same team) or they are transferred to the adult team. In the case of transfer, evaluation together with the receiving physician is required to assess the need to maintain interspersed appointments in the two services until discharge from the Pediatric outpatient clinic.

The pediatrician should act in a preventive manner and identify the concerns of young people and their families that may require changes in the goals of the transition. If there is a failure to achieve the goals set, the plan should be revised, and it may be necessary to increase the frequency of consultations and interventions.
